# The impact of individual and social environmental factors on the health of elderly migrants in China: an analysis based on social-ecological systems theory

**DOI:** 10.1186/s12889-025-23371-2

**Published:** 2025-06-04

**Authors:** Aidi Liao, Jingjin He, Xiafei Tao

**Affiliations:** 1 School of Public Administration, Xiangtan University, Xiangtan, China; 2 School of Government, Shenzhen University, Shenzhen, China

**Keywords:** Elderly Migrants, Individual Factors, Social Environmental Factors, Health, China

## Abstract

**Background:**

With the increasing number of elderly migrants in China, ensuring their health and access to healthcare services have become a significant public health challenge. The social environment of elderly migrants is composed of multiple interconnected systems, each of which exerts a complex and multidimensional influence on their health. This study investigates how individual and social environmental factors affect the health of elderly migrants in China.

**Methods:**

Data from 4,744 respondents were obtained from the 2017 China Migrants Dynamic Survey. Guided by social-ecological systems theory, we explored the relationship between individual and social environmental factors and the health outcomes of elderly migrants using binary logistic regression models.

**Results:**

Individual factors, such as gender, age, education level, and average monthly household income, were found to significantly affect the self-rated health of the respondents. Furthermore, social environmental factors, including types of social interactions, the establishment of health records, the amount of health knowledge acquired, and travel time to medical institutions, also had a notable influence on the health of elderly migrants.

**Conclusions:**

This study suggests that individual and social environmental factors significantly influence the health of elderly migrants. To improve their health, targeted strategies should focus on fostering diverse social networks, enhancing comprehensive health education, optimizing public health services, and creating an equitable institutional framework.

**Supplementary Information:**

The online version contains supplementary material available at 10.1186/s12889-025-23371-2.

## Introduction

Internal migration refers to the movement of individuals within a specific geopolitical unit and is common in many countries and regions worldwide, including the United States, European countries, and China [1, 2]. According to the Seventh National Population Census, the migrant population in China is 376 million, constituting 26.6% of the country’s total population and representing the largest migrant population globally. Approximately 18 million, 7.2% of the total population, are elderly individuals (aged 60 or older) [3, 4]. According to the China Migrants Dynamic Survey, 1 in 10 migrants was aged 60 or older in 2016 [5]. In China, internal migrants are a potentially vulnerable population in terms of health due to the longstanding household registration system (*hukou*) and the dual governance structure that divides rural and urban areas. Most public policies and social benefits are designed and implemented based on the *hukou* system rather than on the actual population residing in a particular area [6, 7]. As a result, elderly migrants are often overlooked and assumed to face no specific health challenges, as they have already surpassed life stages such as reproduction, education, and employment. As a subgroup within both the migrant and elderly populations, they face compounded vulnerabilities due to their dual status, which increases their exposure to health risks.

Research on elderly migrants has examined different groups, including varying age ranges, seasonal migrants, pre-elderly migrants, and those from the baby boomer generation [8–14]. Researchers have extensively examined the factors driving elderly migration, such as changes in family structure, the pursuit of better living conditions (e.g., housing, climate, and living facilities), lower living costs, and improved access to basic public services [15–21]. In China, the main reasons for elderly migration include caring for younger generations, retirement, and participation in work or business activities. Furthermore, the literature often focuses on the consequences of migration, social integration, and elderly migrants’ plans for long-term residence [22–25].

Research on the health of elderly migrants in China generally focuses on three key areas. The first is self-rated health. Elderly migrants tend to report relatively good self-rated health, especially among men and those with higher educational levels. In contrast, older age and being widowed are linked to poorer self-assessment. Elderly migrants with higher monthly household incomes and physically demanding jobs tend to report better health. Moreover, migrants with medical insurance or access to free community-based health screening usually rate their health more positively. However, self-reported health tends to decline with the duration of migration. In addition, elderly migrants who engage in labor or business activities, and those who care for children or grandchildren also tend to report better health [26–30].

Second, the mental health of elderly migrants is an important area of concern. Some scholars argue that their mental health is negatively affected by the stress of migration and the lack of social capital, especially among those who migrate involuntarily. These individuals are not only more likely to experience mental health problems, but also face insufficient support systems for their mental well-being. Other scholars suggest that cultural adaptation stress is just one of several factors, and although elderly migrants may experience a decline in mental health shortly after migration, their condition tends to improve over time. This improvement is influenced by factors such as social interactions, whether they migrate with family or friends, and the availability and quality of community services [31, 32].

Third, healthcare service utilization among elderly migrants is generally lower than that of their native counterparts, a trend observed by both domestic and international researchers [33, 34]. In China, low health awareness and the inability to settle outpatient costs across regions are major factors contributing to low access to healthcare. Approximately 60% of elderly migrants seek medical care outside their place of residence, particularly those from rural areas with shorter residence duration and longer migration distances [35–37].

Although both domestic and international studies highlight the urgent need to focus on elderly migrants'health, research in this area in China remains in its early stages. Previous studies on the health determinants of elderly migrants have primarily focused on individual-level factors that can be controlled or modified, such as marital status, family structure, caregiving arrangements, living conditions, income, education, and diet. In contrast, macro-structural factors, such as institutional frameworks and the allocation of healthcare resources, which individuals have limited ability to influence, have received relatively less attention. When these macro-level factors are considered, they are often examined through the lens of basic public health service utilization, with an emphasis on individual characteristics and behaviors. Therefore, this study, guided by social-ecological theory, analyzes the impact of both individual and social environmental factors on the health status of elderly migrants and provides targeted policy recommendations.

## Analytical framework and research hypotheses

The concept of"ecology"was first introduced by the German biologist Haeckel, who focused on the interactions between organisms and their environment. Over time, ecological perspectives have been applied across fields like public health, psychology, and sociology, leading to theories that highlight the complex environmental influences on individuals. Bronfenbrenner’s social-ecological systems theory, introduced in 1977, proposed a hierarchical structure of environmental influences: the microsystem (immediate environments like family and friends), mesosystem (interactions between microsystems), exosystem (indirect influences like institutions and community networks), and macrosystem (broader cultural, economic, and political contexts). These systems shape behavior and are adaptable in research contexts [38, 39]. Bronfenbrenner did not clearly define the content of each environmental system, emphasizing instead their conceptual scale. Therefore, later researchers often redefine the model based on specific behavioral contexts in which the theory is applied [40, 41].

**Fig. 1:**
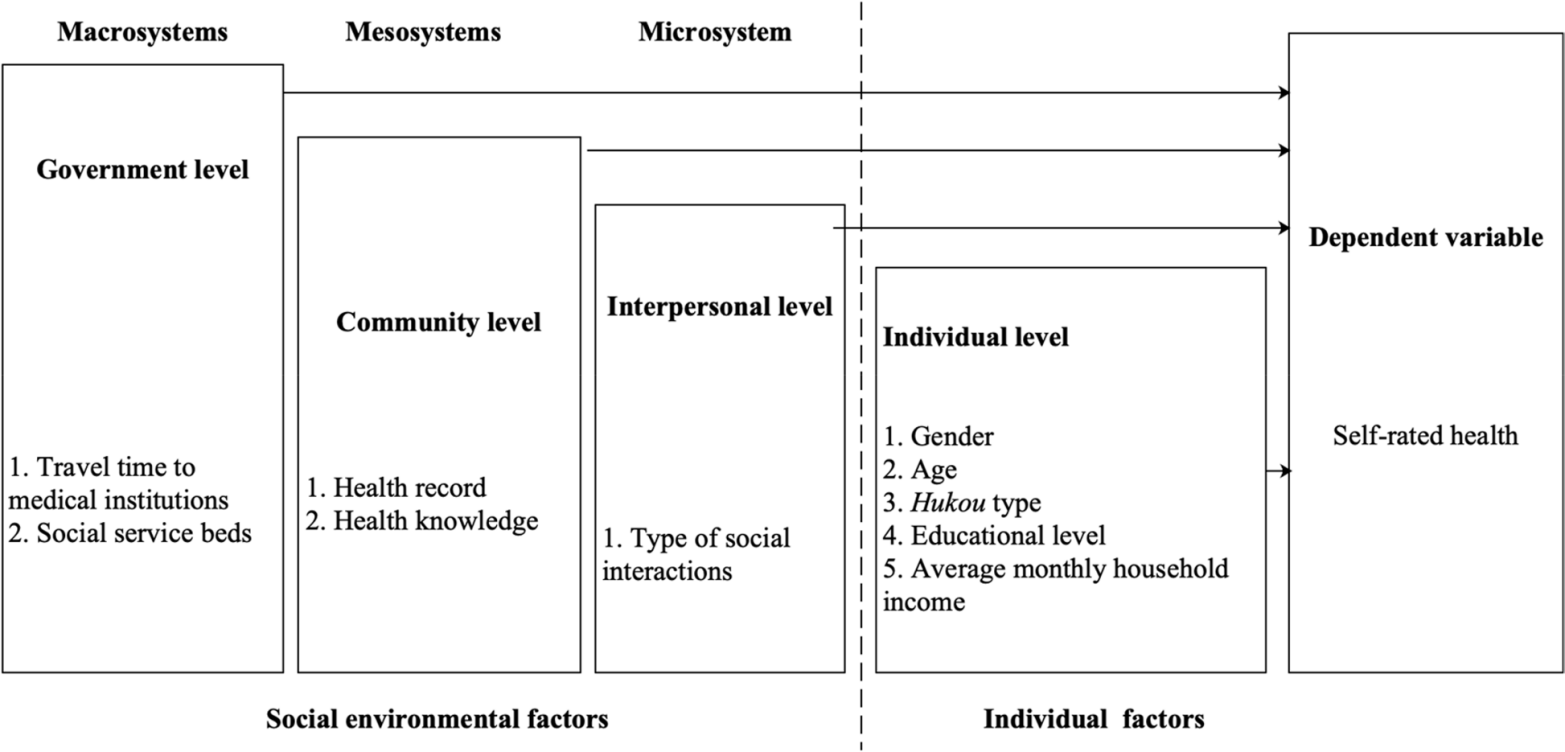
The Social-Ecological Model of Health Influencing Factors for Elderly Migration (SEM-HIFEM)

Based on the social-ecological theory, the social environment of elderly migrants is composed of multiple systems, and their health is inevitably influenced by these systems, making it complex and multidimensional. Considering the characteristics of elderly migrants and the various factors influencing their health, we propose the Social-Ecological Model of Health Influencing Factors for Elderly Migrants (SEM-HIFEM) (Fig. 1). In this model, individual factors primarily encompass demographic and economic characteristics, while social environmental factors are categorized into three levels: the microsystem (interpersonal level), mesosystem (community level), and macrosystem (government level). The microsystem focuses on interpersonal level, such as the number of local friends and marital status. The mesosystem refers to community-level factors, including health records, community-based free health screenings, and the availability of health knowledge. The macrosystem is related to broader government-level factors, such as healthcare insurance and healthcare resources.

Based on the above analysis, we propose two key hypotheses. First, individual factors have a significant impact on the health of elderly migrants. Second, elderly migrants are inevitably influenced by the social environment in which they live.

## Materials and methods

### Data and sample

The data were obtained from the 2017 China Migrants Dynamic Survey (CMDS), organized by the National Health Commission of the People’s Republic of China. This nationally representative annual survey covering all 31 provinces began in 2010, and examines the socio-economic status, health outcomes, and healthcare utilization of migrants. A stratified, multi-stage probability proportional to size sampling method is used for selection. However, the data are only updated through 2018. The 2017 data were chosen for this study due to their extensive coverage of indicators pertaining to the health determinants of elderly migrants (Additional file 1). In total, 159,365 migrants were interviewed. Following the survey’s definition, elderly migrants are individuals aged 60 and above who have resided in the destination area for more than one month without local *hukou*. After excluding 264 cases with missing values, the final sample size of elderly migrants included in this study was 4,744. Notably, 82.07% of these elderly migrants resided in urban areas, aligning with the primary direction of population mobility from rural to urban areas.

### Variable measurement

#### Dependent variables

Self-rated health is a comprehensive measure that effectively predicts individual health outcomes. Its results are relatively objective and show minimal discrepancies when compared to measurements of chronic disease provided by professional institutions, particularly in predicting mortality risk [42–44]. Based on the 2017 CMDS questionnaire, the self-rated health was assessed with the question,'How do you perceive your health?'The response options were very good, good, poor, or very poor. A value of'1'was assigned to responses indicating'good (healthy)'or'very good (basically healthy)', while a value of'0'was assigned to responses indicating'poor (unhealthy but able to take care of myself)'or'very poor (unable to take care of myself).'

#### Independent variables

The independent variables consisted of both individual and social environmental factors. Individual factors included gender, age, *hukou* type, educational level, and average monthly household income(log10-transformed). Social environmental factors included the types of social interactions, health record(yes/no), health knowledge, travel time to medical institutions, and social service beds (per 1,000 people) (see Table 1).

**Table 1:** Definitions and codes for dependent and independent variables

Variable Name	Variable Code
Dependent variables	
Self-rated health	Good (healthy) or Very good (basically healthy) = 1 Poor (unhealthy but able to take care of myself) or Very poor (unable to take care of myself) = 0
Independent variables	
Gender	Male = 1; Female = 0
Age	Continuous variable
*Hukou* type	Agricultural *hukou* = 1; Non-agricultural *hukou* = 0
Education level^1^	Continuous variable
Average monthly household income^2^	Log-transformed
Types of social interactions	Minimal social interaction = 1; Local residents = 2; Non-local residents = 3; Fellow townspeople = 4
Health record	Yes = 1; No = 0
Health knowledge^3^	Continuous variable
Travel time to medical institutions	Less than 15 min = 1; 15 to 30 min = 2; Over 30 min = 3
Social service beds^4^	Continuous variable

Note:

1.Education level was converted from a categorical variable (no primary school education, primary school, junior high school, senior high school/vocational school, and college or above) into a continuous variable by assigning values based on the typical years of schooling: no formal education = 0 years, primary school = 5 years (reflecting the pre-1986 standard prior to the implementation of the Compulsory Education Law of the People's Republic of China, which established a 6-year primary education system), junior high school = 8 years, senior high school/vocational school = 11 years, and college or above = 15 years

2. Average monthly household income was log-transformed to reduce skewness and improve normality in regression analyses

3. Health knowledge was constructed as a continuous variable by calculating the cumulative score derived from responses to the following multiple-choice question:"In the past year, have you received health education in any of the following areas within your current village or community of residence?

4. Social service beds: Given the limited macro-level indicators in the CMDS dataset, the number of social service institution beds from the China Statistical Yearbook 2018 was used as a proxy for healthcare resource availability, using province-level data. This indicator was calculated as the number of beds per 1,000 people by dividing the total number of beds by the year-end total population in 2017. Notably, the China Statistical Yearbook 2018 reports data for 2017, ensuring temporal alignment with the dataset used in this study

### Statistical methods

As self-rated health is a binary variable, we used binary logistic regression models to examine the factors influencing elderly migrants'self-rated health. This model is both easy to interpret and practical, making it well-suited to the analytical needs of the study.

## Results

### Descriptive results

We used Stata 18 for statistical analysis in this study. Table 2 presents the descriptive statistics of the sample, which consisted of 4,744 elderly migrants. The sample was mostly younger elderly migrants (60–74 years): 50.4% aged 60–64 and 27.9% aged 65–69. Most respondents were male (58.6%), married (98.9%), and had an agricultural *hukou* (55.9%). While marital status is typically an important factor in health research, it was not statistically significant in our models, likely due to the very high marriage rate among elderly migrants (98.9%). As a result, marital status was excluded from the subsequent models and was only included for descriptive purposes. The average education level was 6.8 years (SD = 4.0), and the mean monthly household income (log-transformed) was 8.4 (SD = 0.9). In terms of self-rated health, 43.2% of the respondents reported being healthy, while 38.8% described themselves as basically healthy. A smaller proportion of participants considered themselves unhealthy but still capable of independent living (16.9%), and only 1.1% were unable to care for themselves. Notably, 63.6% of elderly migrants had chronic disease.

**Table 2:** Descriptive characteristics of the sample (N = 4,744)

	n	%/M (SD)
Self-rated health		
Healthy	2049	43.2
Basically healthy	1839	38.8
Unhealthy but able to take care of myself	803	16.9
Unable to take care of myself	53	1.1
Chronic disease status		
Yes	3016	63.6
No	1728	36.4
Gender		
Male	2780	58.6
Female	1964	41.4
Age		
60–64	2391	50.4
65–69	1325	27.9
70–74	596	12.6
75–79	294	6.2
80 and above	138	2.9
*Hukou* type		
Agricultural *hukou*	2650	55.9
Non-agricultural *hukou*	2094	44.1
Education level	4744	6.8 (4.0)
Average monthly household income	4744	8.4 (0.9)
Types of social interactions		
Minimal social interaction	1729	36.4
Local residents	1902	40.1
Non-local residents	294	6.2
Fellow townspeople	819	17.3
Marital status		
Married	4691	98.9
Unmarried	53	1.1
Health record		
Yes	1838	38.7
No	2906	61.3
Health knowledge (0–9)	4744	2.7 (3.2)
Travel time to medical institutions		
Less than 15 min	3602	75.9
15 to 30 min	986	20.8
Over 30 min	156	3.3
Social service beds	4744	3.6 (1.6)

*M* mean, *SD* standard deviation

A notable portion of the respondents (40.1%) primarily interacted with local residents, while 36.4% reported minimal social interaction. A smaller group (17.3%) engaged with fellow townspeople, and 6.2% interacted with non-local residents. 38.7% of respondents had established health records, and the average health knowledge (on a scale of 0 to 9) was 2.7 (SD = 3.2). Most respondents (75.9%) reported a travel time to medical institutions of less than 15 min, 20.8% had a travel time between 15 and 30 min, and only 3.3% had to travel more than 30 min. Finally, the average number of social service beds per 1,000 people was 3.6 (SD = 1.6).

### Regression results

We used binary logistic regression to analyze how individual and social environmental factors affected the health of elderly migrants. First, individual factors were included in the model, followed by the incremental inclusion of social environmental variables (Table 3). The multicollinearity analysis in this study showed that the tolerance values ranged from 0.69 to 0.99, all well above the 0.1 threshold, and the variance inflation factors (VIF) ranged from 1.01 to 1.46, with a mean VIF of 1.2, well below the commonly accepted threshold of 10. These results suggest that the likelihood of multicollinearity among the independent variables is minimal.

**Table 3:** Results of model analysis on factors influencing self-rated health

	Model	Model2	Model3	Model4
	b (SE)	b (SE)	b (SE)	b (SE)
**Individual factors**				
**Individual level (a)**				
Gender	0.408^***^ (0.080)	0.401^***^ (0.080)	0.389^***^ (0.082)	0.381^***^ (0.082)
Age	−0.059^***^ (0.006)	−0.057^***^ (0.006)	−0.056^***^ (0.007)	−0.055^***^ (0.007)
*Hukou* type	0.027 (0.093)	0.026 (0.094)	0.074 (0.096)	0.073 (0.096)
Education level	0.063^***^ (0.011)	0.063^***^ (0.011)	0.061^***^ (0.012)	0.059^***^ (0.012)
Average monthly household income	0.456^***^ (0.045)	0.457^***^ (0.046)	0.411^***^ (0.047)	0.397^***^ (0.047)
**Social environmental factors**				
**Interpersonal level (b)**				
Types of social interactions (Reference category: Minimal social interaction)				
Local residents		0.289^***^ (0.086)	0.299^***^ (0.089)	0.306^***^ (0.089)
Non-local residents		0.635^***^ (0.198)	0.588^***^ (0.200)	0.596^***^ (0.201)
Fellow townspeople		0.359^***^ (0.116)	0.303^**^ (0.119)	0.318^***^ (0.120)
**Community level (c)**				
Health record			−0.251^***^ (0.085)	−0.254^***^ (0.087)
Health knowledge			0.063^***^ (0.014)	0.064^***^ (0.014)
**Government level (d)**				
Travel time to medical institutions				−0.332^***^ (0.071)
Social service beds				0.040 (0.027)
_cons	1.064^*^ (0.563)	0.733 (0.568)	0.883 (0.582)	1.243^**^ (0.595)
*N*	5008	5008	4744	4744
pseudo *R*^2^	0.077	0.082	0.084	0.089

*p < 0.1

**p < 0.05

***p < 0.01

In Model 1, which included only individual-level variables, gender, age, education level, and average monthly household income all showed significant associations with self-rated health. Specifically, gender was positively associated with self-rated health (β = 0.408, p < 0.001), indicating that males reported better health than females. Age had a significant negative effect (β = −0.059, p < 0.001), suggesting that older individuals were more likely to report poorer health. Higher education level (β = 0.063, p < 0.001) and average monthly household income (β = 0.456, p < 0.001) were both positively associated with self-rated health, highlighting the importance of socio-economic factors in health perceptions, indicating that individuals with higher education and income levels tended to rate their health more favorably.

In Model 2, we focused on the interpersonal level variables. Social interactions were found to have significant positive associations with self-rated health. Specifically, interacting with other local residents (β = 0.289, p < 0.001) and fellow townspeople (β = 0.359, p < 0.001) was associated with better self-rated health, while interactions with other non-local residents (β = 0.635, p < 0.001) showed an even stronger positive relationship.

In Model 3, the variables at the community level were incorporated. The results revealed that having a health record was negatively associated with self-rated health (β = −0.251, p < 0.001), suggesting that individuals with a health record were more likely to report poorer health. Health knowledge was positively associated with self-rated health (β = 0.063, p < 0.001), indicating that individuals with greater health knowledge tend to rate their health more favorably.

In Model 4, we included the government-level variables. Travel time to medical institutions (β = −0.332, p < 0.001) showed a significant negative association with self-rated health, implying that longer travel times were linked to poorer self-rated health. However, the number of social service beds was not significantly associated with self-rated health (β = 0.040, p > 0.05).

## Robustness test

To ensure the robustness of our findings, we employed two complementary testing methods. First, to mitigate potential measurement errors in the variables, we substituted the dependent variable with chronic disease status, assessed via the question:"Do you have a doctor-diagnosed condition such as hypertension or diabetes?"Second, we applied the Stereotype Logistic Model to account for the ordinal nature of the dependent variable and address the violation of the parallel lines assumption. This model is particularly useful when the categories of an ordinal variable may be indistinguishable. Specifically, Model 1 is the main model, Model 2 uses chronic disease status as the dependent variable, and Model 3 applies the Stereotype Logistic Model. The results across all models were consistent, showing that the key variables maintain their significance levels and directions (see Table 4). Overall, the robustness test results affirm the reliability of our findings and suggest that the conclusions are not sensitive to the choice of dependent variable or model specification.

**Table 4:** Results of robustness test

	(1)	(2)	(3)
	Model1	Model2	Model3
Gender	0.381^***^ (0.082)	0.224^***^ (0.064)	0.580^***^ (0.118)
Age	−0.055^***^ (0.007)	−0.046^***^ (0.006)	−0.113^***^ (0.015)
*Hukou* type	0.073 (0.096)	−0.303^***^ (0.074)	−0.041 (0.121)
Education level	0.059^***^ (0.012)	−0.002 (0.009)	0.070^***^ (0.017)
Average monthly household income	0.397^***^	0.060	0.626^***^
Types of social interactions (Reference category: Minimal social interaction)			
Local residents	0.306^***^ (0.089)	−0.038 (0.070)	0.432^***^ (0.121)
Non-local residents	0.596^***^ (0.201)	0.239^*^ (0.139)	0.696^***^ (0.243)
Fellow townspeople	0.318^***^ (0.120)	0.107 (0.093)	0.427^***^ (0.154)
Health record	−0.254^***^ (0.087)	−0.288^***^ (0.067)	−0.287^**^ (0.112)
Health knowledge	0.064^***^ (0.014)	0.026^**^ (0.010)	0.073^***^ (0.018)
Travel time to medical institutions	−0.332^***^ (0.071)	−0.029 (0.060)	−0.477^***^ (0.106)
Social service beds	0.040 (0.027)	0.022 (0.020)	0.068^**^ (0.034)
_cons	1.243^**^ (0.595)	3.401^***^ (0.496)	
phi1_1_cons			0.840^***^ (0.093)
phi1_2_cons			0.386^***^ (0.049)
theta1_cons			−5.290^***^ (0.858)
Theta2_cons			−2.233^***^ (0.677)
Theta3_cons			−0.581^*^ (0.324)
*N*	4744	4744	4744
pseudo *R*^2^	0.089	0.026	

*p < 0.1,

**p < 0.05

***p < 0.01

## Discussion

### Individual factors

The study revealed significant gender disparities in self-rated health among elderly migrants, with female respondents reporting poorer outcomes than males—a pattern consistent with existing literature on gendered health inequalities [45–49]. As suggested by Liu Yuzhi, male elderly individuals generally have higher educational attainment than their female counterparts, which may contribute to their persistently better self-rated health and fewer reported disease symptoms [50].This divergence may stem from biological vulnerabilities, sociocultural roles, and differential access to healthcare resources. Furthermore, we found self-rated health progressively declined with age, aligning with natural physiological deterioration [51, 52]. Notably, this study suggested *hukou* type, demonstrated no significant association with health outcomes, reflecting the complex interplay between rural–urban health dynamics. While rural residents may experience better physical health, urban migrants often face mental health challenges due to acculturative stress [53, 54].

In addition, this study indicated that educational attainment emerged as a robust predictor of health status. Higher education correlates with enhanced employment opportunities, socioeconomic stability, and health literacy, enabling proactive health investments and healthier lifestyle choices [55, 56]. Grossman M posits that educated individuals demonstrate a greater propensity to adopt preventive health behaviors (e.g., maintaining balanced nutrition and engaging in regular exercise) while avoiding risk factors (e.g., tobacco use), consequently attaining enhanced physical and psychological well-being [57]. Furthermore, we found socioeconomic status, proxied by household income, further amplified these benefits. This is because affluent families can secure quality housing, nutrition, and leisure activities while mitigating financial barriers to healthcare access—critical factors in contexts with fragmented medical insurance systems [58–60]. Elevated income also facilitates timely medical interventions, reducing health deterioration risks from delayed treatment.

### Social environmental factors

Our study also found social engagement significantly enhanced self-rated health among elderly migrants, as evidenced by comparative analyses between socially active and isolated groups [61, 62]. Interactions with both local and non-local networks provide dual benefits: localized ties improve healthcare navigation and psychological adaptation, while cross-regional connections offer cultural enrichment and cognitive stimulation, fostering resilience [63–65]. Conversely, social isolation exacerbates depression risks and deteriorates mental health [66].

A counterintuitive finding concerned the negative association between health record ownership and self-rated health. Typically, studies suggest that electronic health records facilitate the collection of health data and capture social determinants of health, thereby promoting population health [67–69]. This finding in our study may anomaly reflect China’s policy-driven emphasis on health record quotas, which incentivizes perfunctory documentation by overburdened local staff, primarily targeting already ill populations [70]. In contrast, we found health knowledge acquisition positively correlated with self-rated health by empowering individuals to adopt preventive behaviors and manage chronic conditions [71–76].

At the government level, this study suggested healthcare accessibility, measured by travel time to medical facilities, emerged as a critical determinant of health outcomes. Proximity to healthcare services exerts a stronger influence on health outcomes than technological advancements, especially for elderly migrants who experience mobility constraints [76–81]. However, destination-area social service bed availability in this study showed negligible impact, likely due to systemic exclusion mechanisms (e.g., hukou-based eligibility, insurance portability barriers) that restrict migrants’ utilization of local resources [34, 82, 83]. Wang Q's study revealed that elderly migrants derive limited benefits from public medical services, yet their health status demonstrates a positive correlation with local economic development. Increased disposable income resulting from destination-area economic growth enhances health investment capacity, contingent upon such economic gains being effectively translated into household income [84].

However, due to the complexity of factors influencing health, it is not feasible to cover all potential variables in a single study. This research focuses on the most significant aspects of the key issues, and although efforts were made to account for as many health determinants as possible, the available indicators remain limited. Additionally, since the data of CMDS are cross-sectional, we cannot make a valid causal inference or control for unobserved heterogeneity based on such data. Furthermore, the use of 2017 CMDS data may introduce some temporal lag. Nonetheless, considering the relative stability of the elderly migrant, the conclusions still hold considerable relevance.

## Conclusions

This study explored the impact of individual and social environmental factors on the self-rated health of elderly migrants in China. The findings highlighted individual factors, such as gender, age, education level, and average monthly household income, significantly influenced the self-rated health of elderly migrants. Social environmental factors, such as social interactions, health record, health knowledge, and travel time to medical institutions, also played a role. Interestingly, the number of social service beds (per 1,000 people) did not significantly affect health outcomes. These results suggest that both individual characteristics and social environments contribute to the health disparities faced by elderly migrants. The study underscores the need for targeted strategies, such as fostering diverse social networks, enhancing comprehensive health education, optimizing public health services, and creating an equitable institutional framework.

## Supplementary Information

Additional file 1. Comparison of key indicators from CMDS.  

## Data Availability

The datasets used in this study are publicly available and can be accessed via the Migrant Population Service Center, National Health Commission of China. Requests to access these datasets should be directed to https://www.chinaldrk.org.cn/wjw/#/home.
