# Supplementary material for: The impact of individual and social environmental factors on the health of elderly migrants in China: an analysis based on social-ecological systems theory
Source: BMC Public Health. 2025 Jun 4;25:2091. doi: 10.1186/s12889-025-23371-2 (PMC12139264; doi:10.1186/s12889-025-23371-2)
Supplement: Supplementary file 1 — Additional file 1. Comparison of key indicators from CMDS. [file 12889_2025_23371_MOESM1_ESM.docx]

Additional file 1: Comparison of key indicators from CMDS

| **Indicators** | **2015** | **2016** | **2017** | **2018** |
| --- | --- | --- | --- | --- |
| Gender | 🗸 | 🗸 | 🗸 | 🗸 |
| Age | 🗸 | 🗸 | 🗸 | 🗸 |
| *Hukou* type | 🗸 | 🗸 | 🗸 | 🗸 |
| Education level | 🗸 | 🗸 | 🗸 | 🗸 |
| Primary source of income | 🗸 |  |  |  |
| Marital status | 🗸 | 🗸 | 🗸 | 🗸 |
| Average monthly household income (RMB) | 🗸 | 🗸 | 🗸 | 🗸 |
| Reasons for migration | 🗸 |  | 🗸 | 🗸 |
| Duration of migration (years) | 🗸 |  | 🗸 | 🗸 |
| Migration area | 🗸 |  | 🗸 | 🗸 |
| Exercise duration (hours) | 🗸 |  |  |  |
| Number of local friends | 🗸 |  |  |  |
| Communication partner |  |  | 🗸 |  |
| Health record | 🗸 | 🗸 | 🗸 | 🗸 |
| Time to access healthcare services |  |  | 🗸 |  |
| Community-based free health screening | 🗸 |  |  |  |
| Health knowledge | 🗸 | 🗸 | 🗸 | 🗸 |
| Health Insurance | 🗸 | 🗸 | 🗸 | 🗸 |
| Self-rated health | 🗸 |  | 🗸 | 🗸 |
| Chronic diseases | 🗸 |  | 🗸 |  |
